# A comparison of running kinetics in children with and without genu varus: A cross sectional study

**DOI:** 10.1371/journal.pone.0185057

**Published:** 2017-09-19

**Authors:** Amir Ali Jafarnezhadgero, Morteza Madadi Shad, Mahdi Majlesi, Urs Granacher

**Affiliations:** 1 Department of Physical Education and Sport Sciences, University of Mohaghegh Ardabili, Ardabil, Iran; 2 Department of Sport Biomechanics, Faculty of Humanities, Islamic Azad University, Hamedan Branch, Hamedan, Iran; 3 Division of Training and Movement Sciences, Research Focus Cognition Sciences, University of Potsdam, Potsdam, Germany; Nanyang Technological University, SINGAPORE

## Abstract

**Introduction:**

Varus knee alignment has been identified as a risk factor for the progression of medial knee osteoarthritis. However, the underlying mechanisms have not been elucidated yet in children. Thus, the aims of the present study were to examine differences in ground reaction forces, loading rate, impulses, and free moment values during running in children with and without genu varus.

**Methods:**

Thirty-six boys aged 9–14 volunteered to participate in this study. They were divided in two age-matched groups (genu varus versus healthy controls). Body weight adjusted three dimensional kinetic data (Fx, Fy, Fz) were collected during running at preferred speed using two Kistler force plates for the dominant and non-dominant limb.

**Results:**

Individuals with knee genu varus produced significantly higher (*p* = .01; *d* = 1.09; 95%) body weight adjusted ground reaction forces in the lateral direction (Fx) of the dominant limb compared to controls. On the non-dominant limb, genu varus patients showed significantly higher body weight adjusted ground reaction forces values in the lateral (*p* = .01; *d* = 1.08; 86%) and medial (*p* < .001; *d* = 1.55; 102%) directions (Fx). Further, genu varus patients demonstrated 55% and 36% greater body weight adjusted loading rates in the dominant (*p* < .001; *d* = 2.09) and non-dominant (*p <* .*001*; *d* = 1.02) leg, respectively. No significant between-group differences were observed for adjusted free moment values (*p*>.05).

**Discussion:**

Higher mediolateral ground reaction forces and vertical loading rate amplitudes in boys with genu varus during running at preferred running speed may accelerate the development of progressive joint degeneration in terms of the age at knee osteoarthritis onset. Therefore, practitioners and therapists are advised to conduct balance and strength training programs to improve lower limb alignment and mediolateral control during dynamic movements.

## Introduction

The knee joint is particularly vulnerable for running injuries with a prevalence rate of 40% affecting the knee joint [1]. Knee injuries are often encountered in child and adolescent sports [2] and thus represent a meaningful cause for the later development of osteoarthritis) OA) [3]. Even though OA is a chronic disabling disorder usually occurring in elderly individuals, the number of children and adolescents suffering from OA has dramatically increased globally over the past decades [3]. It has been proposed that an impaired ability to control lower limb alignment during the performance of activities of daily living due to for instance muscle weakness is associated with mechanical stress within the knee joint [4]. In children with genu varus, higher contact stresses on the knee joints represent one of the main causes associated with medial OA development [5,6]. These contact stresses and thus the risk of developing knee OA are even increased if youth with genu varus perform weight-bearing exercises [7–10].

Previous studies have examined children with genu varus compared to healthy controls while walking and reported between-group differences in sagittal, frontal, and horizontal kinematics and kinetics. For example, it was demonstrated that knee varus malalignment resulted in larger endorotation of the foot and greater internal tibia rotation during the stance phase of walking [5]. In addition, significant and high associations were reported between the maximum external knee adduction moment and internal knee rotation during the terminal stance phase of walking in genu varus children [5]. Running compared to walking is biomechanically (e.g., higher impact forces) and physiologically (e.g., neuromuscular and cardiovascular) more demanding for genu varus patients because running produces higher compressive loads on the knee joint [11].

To the authors’ knowledge, there is no study available in the literature that examined kinetics during running in children with genu varus compared to health young controls. Therefore, it is timely and imperative to assess lower limb loads during children’s everyday activities such as running. Knowledge on this research question can be used to determine key risk factors associated with OA progression in youth patients. While there is evidence that long-term and high-volume running training may induce degenerative changes to the articular cartilage resulting in progression of knee OA [12,13], less is known on the relationship between static knee varus malalignment and three dimensional ground reaction forces (GRF) during running in children with genu varus.

Thus, the aims of the present study were to examine differences in three dimensional GRF (Fx, Fy, Fz) during running at preferred speed in boys with genu varus compared to age-matched healthy controls. Additionally, we were interested in studying between-group differences during running in time to reach peak (TTP) GRF, vertical loading rates, impulses in all axes, and free moment (FM) of the dominant and non-dominant lower limbs. With reference to the relevant literature [5,6], we hypothesized that the vertical (Fz) and mediolateral (Fx) GRF during running is higher in children with genu varus compared to their healthy peers. In addition, we expected higher loading rates, mediolateral impulses, and FM in children with genu varus compared to healthy controls which is indicative of an altered neuromuscular activation strategy [5,14].

## Material and methods

### Participants

**Fig 1:**
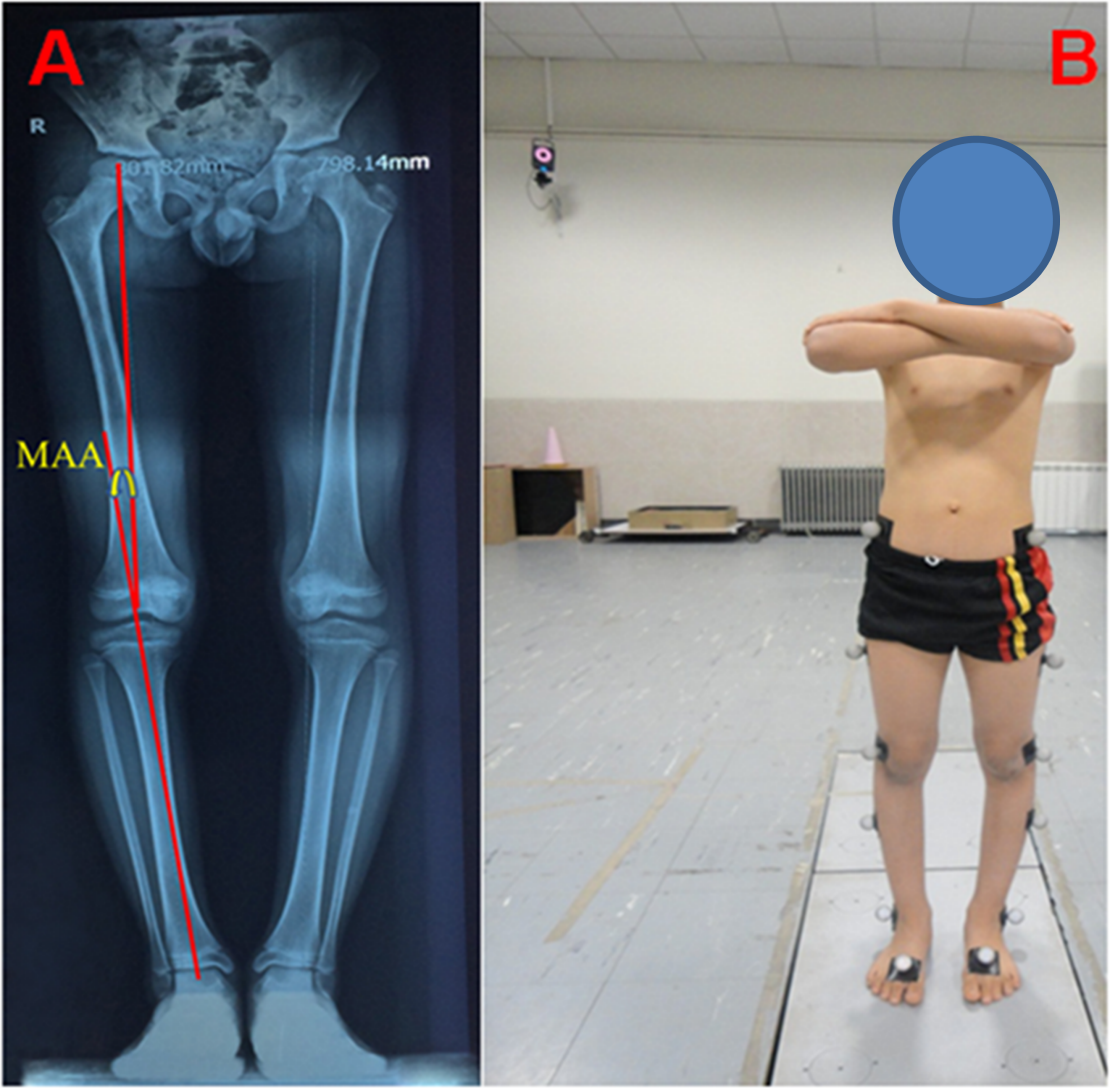
The view of (A) mechanical axis angle (MAA), and (B) plugin gait marker set. Written informed consent (as outlined in the PLOS consent form) was obtained from the parents of the child in Fig 1. All participants provided their written informed consent/assent.

We used the freeware tool G*Power (http://www.gpower.hhu.de/) to calculate an a priori power analysis with the test family (t tests) and the respective statistical test (means: difference between two independent means [two groups]) based on a related study that examined between-group differences in running kinetics (i.e., GRF) in injured and uninjured runners [15]. The power analysis was computed with an assumed Type I error of 0.05, a Type II error rate of 0.20 (80% statistical power) and an effect size of 0.80 for active running kinetics. The analysis revealed that 36 children would be sufficient to observe large between group differences. Thirty-six boys aged 9–14 were enrolled in this study. Participants were divided in two groups (genu varus and healthy controls). The genu varus group included 18 boys that were selected during clinical examinations (Table 1). Only patients were enrolled that had a mechanical axis angle (MAA) which was defined as the angle formed by straight lines drawn from the centre of the hip to the centre of the knee and the centre of the knee to the centre of the ankle [16] greater than 1.3° in both knees. This was determined by means of a full-length standing anteroposterior radiograph [17] (Fig 1). Exclusion criteria were signs of joints diseases, diseases of bones, ligament injury, neuromuscular dysfunction, diseases of tendon, history of major trauma or surgery of the lower extremities, chronic joint infection, intra articular corticosteroid injection [5]. All participants were right lower-limb dominant as determined by a kicking ball test [18]. In addition, kinematic data was used to detect footfall patterns (heel strike pattern instead of mid foot or forefoot strike) during running test trials to match our participants in the two experimental groups not only for limb dominance but also for similar footfall characteristics. Table 1 illustrates group characteristics. It was previously postulated that shoe type affects walking kinetics in children [19,20] which is why all participants of the present study were equipped with individually fitted (i.e., size) athletic shoes (New Balance 759, USA) during running.

**Table 1:** Group-specific characteristics of our participants presented as means and standard deviations.

Variable	Healthy controls (n = 18)	Genu Varus (n = 18)	p
Age (years)	11.44±1.78	11.66±1.64	0.70
Body height (m)	1.40±0.07	1.40±0.08	0.95
Body mass (kg)	36.39±12.13	36.55±11.44	0.97
BMI (kg/m^2^)	18.08±4.47	18.13±4.04	0.97
Dominant MAA	-	9.5±6.1	N/A
Non-dominant MAA	-	9.2±5.3	N/A

Note. MAA = Mechanical axis angle; BMI = Body mass index; N/A, Not applicable; p value from independent samples t-test.

All participants and their parents or legal representatives provided written informed consent/assent to participate in this study. This study was approved by the local ethics committee (University of Mohaghegh Ardabili, Iran) and all experiments were conducted in accordance with the latest version of the Helsinki Declaration.

### Running analysis

This cross-sectional study included boys aged 9–14 with genu varus and healthy controls. Our participants were recruited from local physical therapy clinics. Three-dimensional running analysis was performed using a Vicon MX system consisting of six T- series cameras (200 Hz) (Vicon Motion Systems, Oxford, UK). Two Force plates (Kistler AG, Winterthur, Switzerland) embedded in the middle of an 18-m walkway simultaneously recorded three dimensional GRF data (Fx, Fy, Fz) at a sampling frequency of 1000 Hz. A plug-in-gait marker set [21] with sixteen reflective markers (14 mm diameter) was used to identify the pelvis, thighs, legs, and feet segments. Marker positioning was identified through palpation and in accordance with guidelines provided by the manufacturer. The markers were directly attached to the skin of the following anatomical landmarks: bilateral anterior and posterior superior iliac spines; bilateral lateral femoral epicondyles; bilateral lateral malleoli; bilateral calcaneus; lateral side of the thighs and shanks, and top of the feet at the base of the second metatarsal.

Thereafter, participants were familiarized with the laboratory environment and walkway area and at least five practice trials were performed to ensure subjects were able to strike the force plate without consciously changing their running cadence. Afterwards, each participant identified with heel strike pattern during running (kinematic analysis) performed five acceptable shod running trials at preferred and comfortable speed. A trial was discarded if both feet did not land on the force plates, if the participant targeted the platforms, lost balance during the trial, ran with mid or forefoot strike pattern, or even fell during running. Kinematic and kinetic data were extracted during the stance phase of running, defined as the interval from ground contact (onset of vertical GRF [Fz] > 10 N) to toe off (vertical GRF [Fz] < 10 N) [22,23]. Kinematic and kinetic data were filtered using a fourth-order low-pass Butterworth filter with a cutoff frequency of 10 and 20 Hz, respectively. Using spline interpolation, all data were normalized to the stance phase with heel contact to toe-off corresponding to 100% [24].

**Fig 2:**
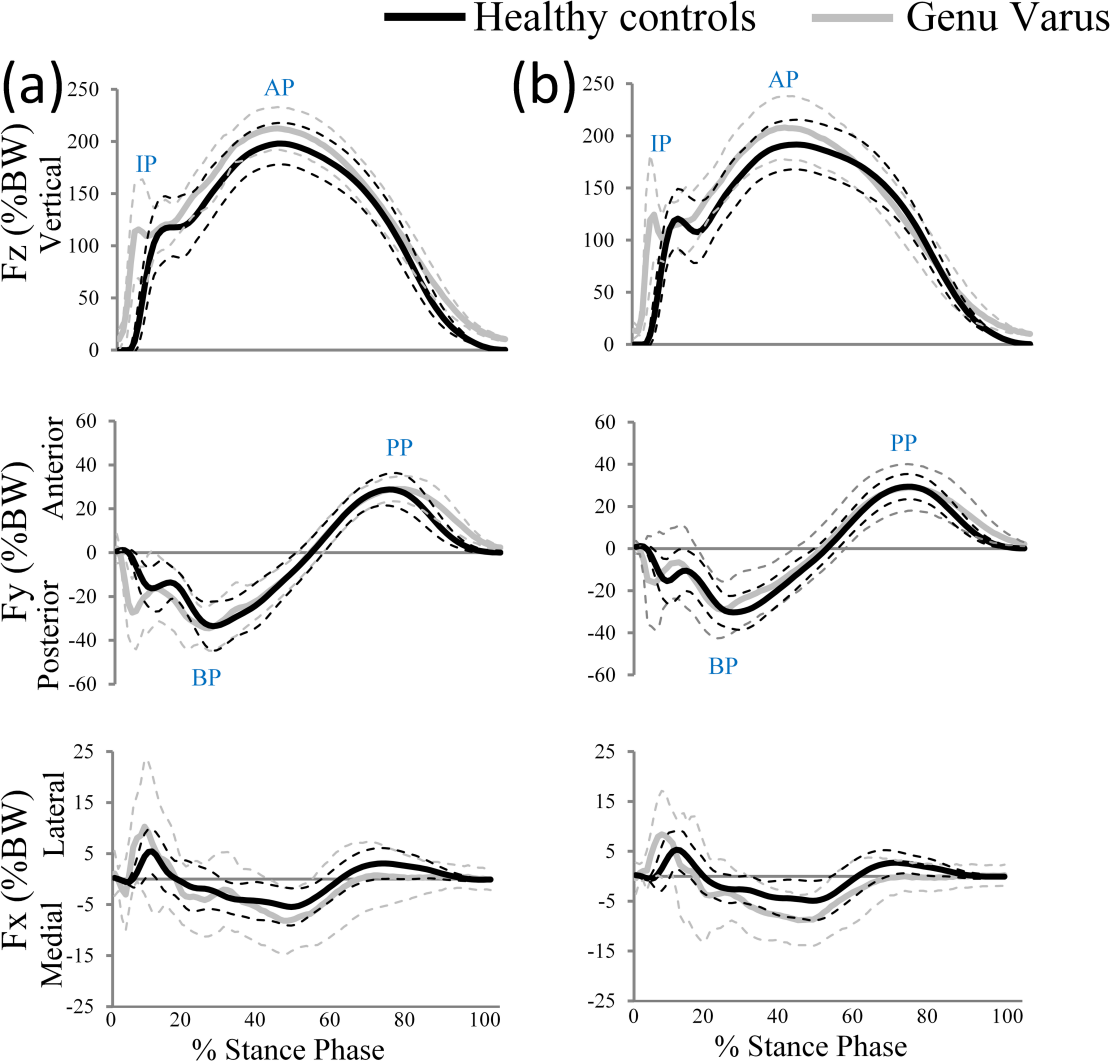
Time-normalized traces of the ground reaction forces for both groups during the stance phase of a running cycle. Reaction forces (Fx, Fy, Fz) are demonstrated for the (a) dominant and (b) non-dominant lower limbs. “IP”, “AP”, “BP” and “PP” represent abbreviations that stand for “Impact Peak”, “Active Peak”, “Braking Peak” and “Propulsion Peak”, respectively. The solid lines represent the mean values, whereas the dashed lines indicate standard deviation values.

Parameters that were used for further statistical analyses included peak values in three dimensional GRF and time to peak of the mediolateral (Fx), anteroposterior (Fy), and vertical (Fz) GRF axes. The first and second peaks of the bimodal vertical GRF curve (impact peak [IP] and active peak [AP]) were considered for further analysis (Fig 2). In the mediolateral direction, the peaks of lateral force and medial force were also analyzed (Fig 2). In the anteroposterior direction, the braking peak (BP) and propulsion peak (PP) were analyzed (Fig 2). Forces were normalized to body weight (BW) and the corresponding timing was expressed as a percentage of stance-phase duration (%Stance), to allow between-subject comparison [25].

Vertical loading rates were computed as the average slope from 20% to 80% of the vertical GRF (Fz) at the point of interest [26]. Impulses were calculated using the trapezoidal integration method (Eq 1) for x, y, and z axes as follows [27]:

$$\mathrm{Impulse}=\Delta t\left[\left(\frac{F1+Fn}{2}\right)+\sum_{i=2}^{n-1}Fi\right]$$ (Eq 1)

Where, F_1_ and F_n_ are the first and last forces, respectively; Δt is equal to sampling duration and n is the number of force samples.

The FM was calculated by using the following equation [28]:

FM=Mz−Fy(CoPx)+Fx(CoPy) (Eq 2)

Where M_z_ is the moment around the force plate vertical axis, CoP_x_ and CoP_y_ are the locations of the center of pressure (CoP) along the mediolateral and anteroposterior axes, respectively. All FM waveforms were amplitude-normalized to the product of each individual's body weight (N) and height (m) [28]. The first peak of the FM curve (negative; abductor moment) and the second peak (positive; adductor moment) were included in our analyses. Impulses of FM were computed as the net area under the FM curve during stance [29].

### Statistical analyses

Data were tested for normal distribution using the Shapiro-Wilk test. Homogeneity of variance was assessed using the Levene’s test and variance ratios. Data were presented in the form of means and standard deviations if normal distribution of data was given. Between-group differences were tested for significance using the independent samples t-test. The significance level was set at *p*<0.01. In addition, effect sizes (Cohen’s *d*) were calculated as a ratio of mean difference divided by the mean standard deviation of both groups. Effect sizes were considered small if *d* < 0.5, medium if 0.5 ≤ *d* < 0.8 and large with *d* ≥ 0.8 [30]. The statistical calculations were carried out using the SPSS version 16 (Chicago, IL, USA).

## Results

Our statistical analysis did not detect any significant between-group difference in running speed. Running speed of the genu varus group was 3.23±0.14 m/s and 3.26±0.15 m/s for the healthy control group (*p*>0.05, CI: -0.09, 0.07).

Table 2 compares mean and body weight adjusted peak GRF values between groups for the dominant and non-dominant lower limbs during the stance phase of a running cycle. In the dominant lower limb, individuals with knee genu varus attained 95% higher GRF values in the lateral direction (Fx) compared to healthy controls (*p* = .01, *d* = 1.09). Similarly, they showed 86% higher GRF values in the lateral direction (Fx) (*p* = .01, *d* = 1.08) as well as 102% higher GRF values in the medial direction (Fx) (*p* < .001, *d* = 1.55) of the non-dominant lower limb.

**Table 2:** Means and standard deviations of peak GRF values in vertical (Fz), anterior-posterior (Fy) and mediolateral (Fx) directions for both groups. Data were normalized to each individual’s body weight (%BW).

Side	Variable	Component	Group		p value	Effect Size (d)	95% CI of difference
			Healthy controls	Genu Varus			
Dominant	Fz	Impact	131.5±27.1	132.8±56.3	0.93	0.03	-31.7, 28.9
		Active	200.2±20.1	216.8±18.8	0.02	0.85	-29.7, -3.3
	Fy	Braking	34.8±10.9	43.9±12.9	0.03	0.76	0.9, 17.1
		Propulsion	29.1±7.6	30.2±5.9	0.63	0.16	-5.7, 3.5
	Fx	Lateral	7.7±3.1	14.9±10.2	0.01*	1.09	-12.6, -2.1
		Medial	7.0±2.8	11.6±6.4	0.01	0.49	1.1, 7.9
Non-dominant	Fz	Impact	127.6±31.9	144.9±39.8	0.16	0.48	-41.5, 6.9
		Active	194.1±23.4	213.3±26.9	0.03	0.38	-36.3, 2.1
	Fy	Braking	31.8±8.3	38.4±17.3	0.16	0.51	-2.7, 15.9
		Propulsion	29.7±6.0	30.4±11.1	0.82	0.08	-6.8, 5.4
	Fx	Lateral	7.3±3.8	13.7±7.9	0.01*	1.08	-10.6, -2.1
		Medial	6.4±3.4	13.1±5.2	< .001*	1.55	3.7, 9.6

* Note. Significant between-group differences.

Fig 2 illustrates the patterns of GRF in all directions for both legs. The graph indicates that subjects from both groups showed similar patterns in anterior–posterior direction (Fy) but different patterns in other planes of motion. In particular, genu varus patients showed significantly greater peak GRF values in medial and lateral directions (Table 2).

Table 3 presents the TTP GRF values for both groups. Overall, the healthy control group showed significantly longer times to reach peak GRF values (except TTP for the propulsion peak) compared to genu varus patients. In the dominant limb, a significantly longer duration in time to reach peak GRF was found for impact (Fz) (*p* < .001, *d* = 3.72, 150%) and the posterior braking (Fy) peak GRF (*p* < .001, *d* = 1.30, 47%). Likewise, in the non-dominant lower limb, our analysis revealed 111% and 42% longer duration to reach the vertical GRF impact (Fz) (*p* < .001, *d* = 3.68) and the posterior braking peak GRF (Fy) (*p* = .01, *d* = 1.08), respectively.

**Table 3:** Means and standard deviations of time to reach peak GRF components in vertical (Fz), anterior-posterior (Fy) and mediolateral (Fx) directions for both groups. Times are expressed as a percentage of the stance-phase duration.

Side	Variable	Component	Healthy controls (% Stance)	Genu Varus (% Stance)	p value	Effect Size (d)	95% CI of difference
Dominant	Fz	Impact	12.4±2.6	4.9±1.4	< .001*	3.72	6.0, 8.9
		Active	42.3±4.8	41.9±5.0	0.84	0.06	-2.9, 3.6
	Fy	Braking	24.4±6.1	16.4±9.1	< .001*	1.30	2.7, 13.2
		Propulsion	71.7±1.8	74.5±4.4	0.02	0.93	-5.2, -0.6
	Fx	Lateral	24.1±22.9	11.0±6.3	0.03	0.89	1.4, 24.7
		Medial	39.2±16.2	34.2±24.6	0.48	0.24	-9.2, 19.1
Non-dominant	Fz	Impact	11.6±2.0	5.5±1.3	< .001*	3.68	4.9, 7.3
		Active	41.0±6.2	40.7±4.9	0.86	0.07	-3.5, 4.1
	Fy	Braking	24.8±4.3	17.4±9.4	0.01*	1.08	2.4, 12.4
		Propulsion	71.3±1.9	72.7±5.3	0.29	0.20	-4.2, 1.3
	Fx	Lateral	15.5±13.5	8.5±3.6	0.04	0.81	0.3, 13.7
		Medial	42.2±13.4	34.2±15.9	0.12	0.54	-2.0, 17.9

* Note. Significant between-group differences.

**Fig 3:**
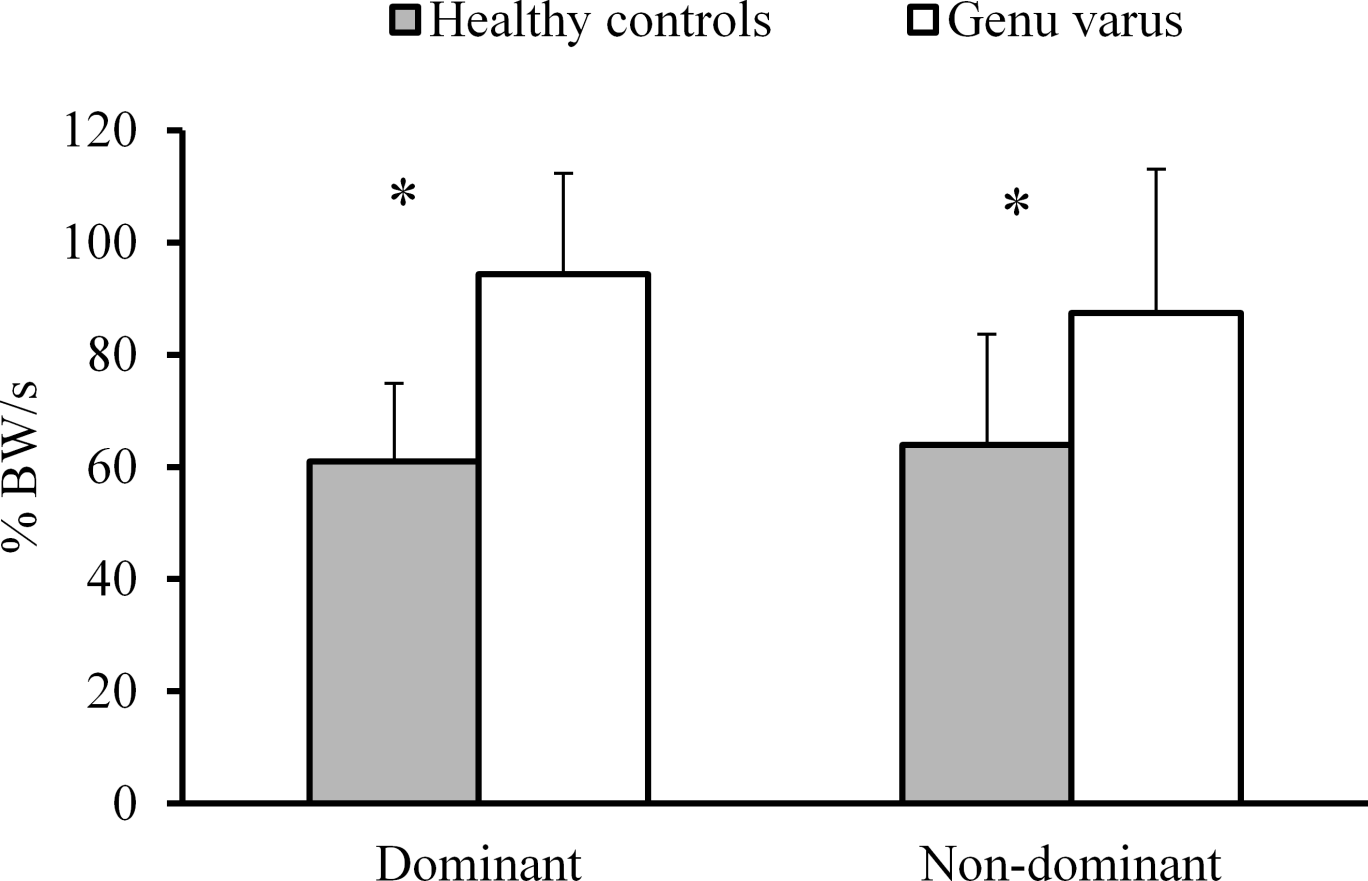
Vertical loading rate values during the stance phase of a running cycle.

Fig 3 illustrates between-group differences in vertical loading rates for both, the dominant and non-dominant lower limbs during the stance phase of a running cycle. The genu varus group demonstrated greater loading rate in the dominant (*p* < .001, *d* = 2.09, 55%, CI: -44.3, -22.5) and non-dominant (*p* < .001, *d* = 1.02, 36%, CI: -39.0, -7.8) lower limbs, respectively.

**Fig 4:**
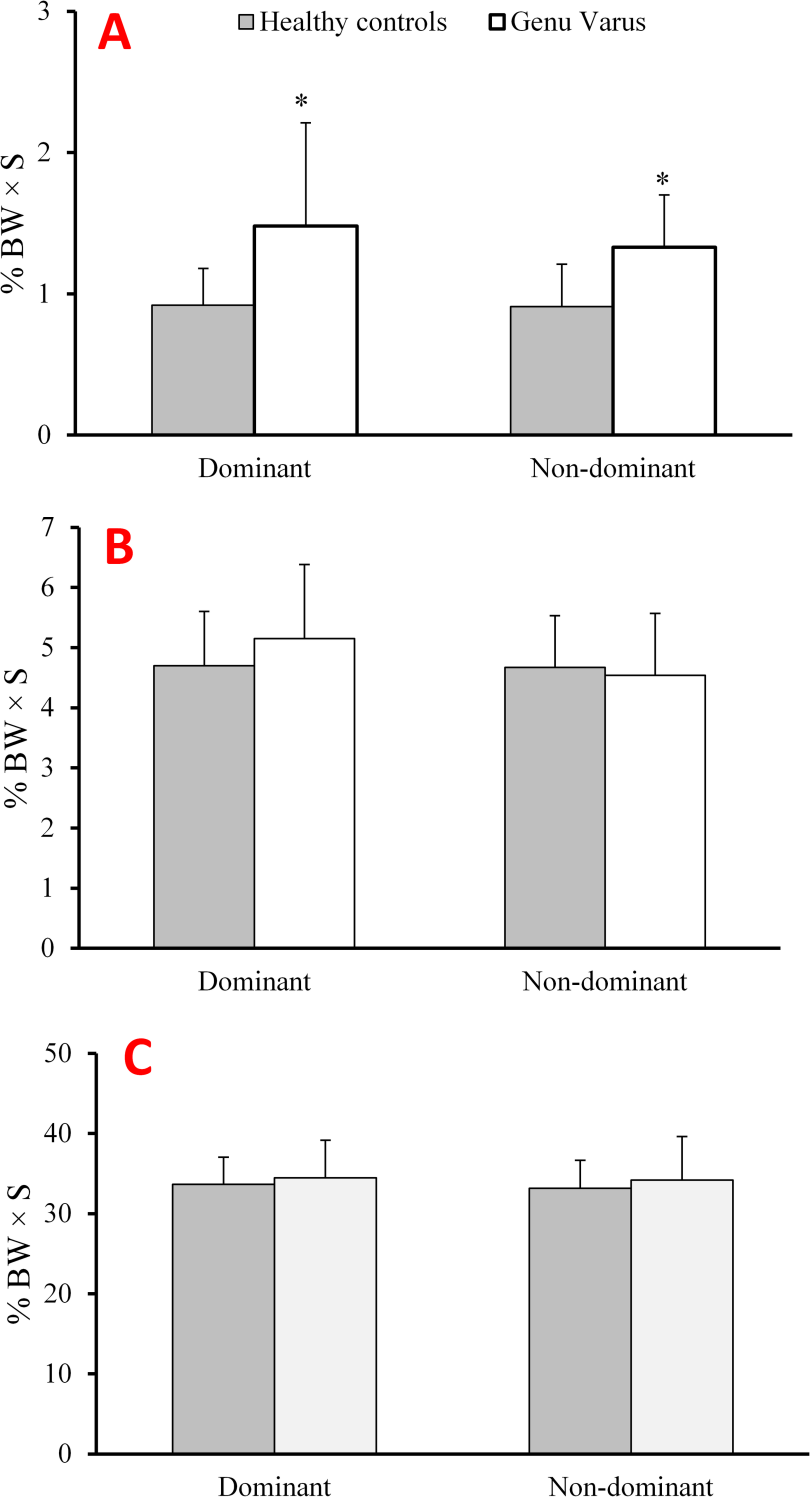
Means and standard deviations of (a) impulse x, (b) impulse y, and (c) impulse z values for both groups during the stance phase of a running cycle.

With regards to the impulse amplitude, genu varus patients showed significantly higher values in the mediolateral direction for both, the dominant (*p* = .01; *d* = 1.12, 60%, CI: -0.9, -0.2) and non-dominant (*p* < .001, *d* = 1.24, 46%, CI: -.7, -0.2) lower limbs as compared to controls (Fig 4).

**Fig 5:**
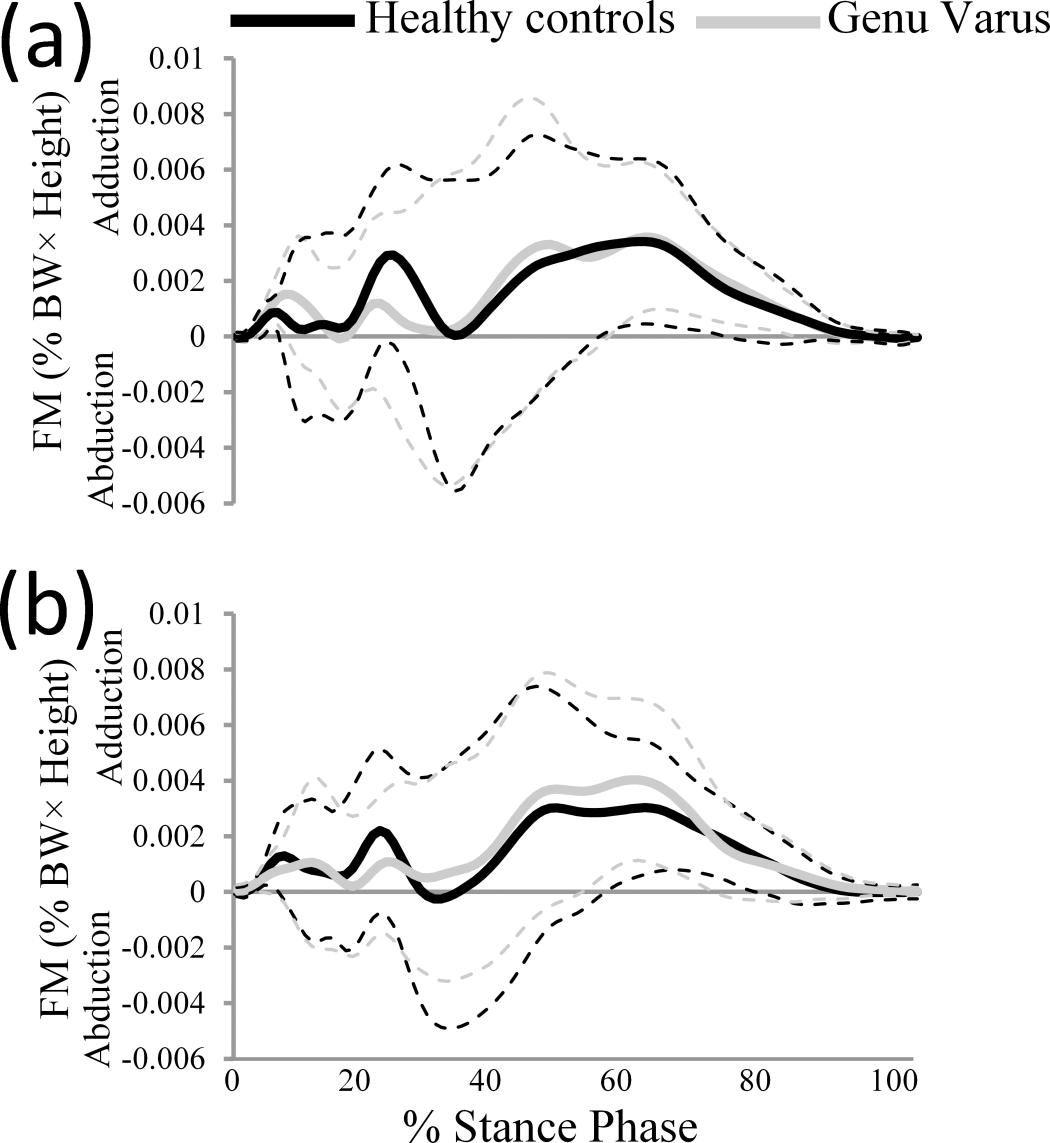
Time-normalized traces of the free moment for both groups during the stance phase of a running cycle. Free moments for the (a) dominant and (b) non-dominant lower limbs are displayed.

Table 4. indicates no significant between-group differences in terms of body-weight adjusted free moments. Fig 5 illustrates the patterns of free moments for the dominant (a) and non-dominant (b) lower limbs during the stance phase of a running cycle.

**Table 4:** Averaged and normalized (% body weight × height) free moment variables in healthy controls and genu varus children.

Side	Variable	Component	Group		p value	Effect Size (d)	95% CI of difference
			Healthy controls	Genu Varus			
Dominant	Free Moment	Peak Adduction	5.4±3.1	5.7±3.5	0.83	0.07	-2.4, 2.0
		Impulse	1.4±1.9	1.4±1.9	0.99	0.01	-1.3, 1.3
Non-dominant	Free Moment	Peak Adduction	5.0±3.2	5.9±3.3	0.46	0.25	-3.0, 1.3
		Impulse	1.3±1.8	1.5±1.7	0.71	0.13	-1.4, 0.9

Note. Petak Adduction values are × 10^−3^; Impulse values are × 10^−4^.

## Discussion

This is the first study that examined three dimensional running GRF in boys with genu varus compared to healthy and age-matched controls. We hypothesized that body weight adjusted vertical (Fz) and mediolateral (Fx) GRF during running is higher in children with genu varus compared to their healthy peers. In addition, we expected higher body weight adjusted loading rates, mediolateral impulses, and FM in children with genu varus compared to healthy controls.

The most important findings of this study were that individuals with knee genu varus produced significantly higher body weight adjusted GRF values in the lateral direction (Fx) of the dominant limb compared to controls. On the non-dominant limb, genu varus patients showed significantly higher body weight adjusted GRF values in the lateral and medial directions (Fx). Further, genu varus patients demonstrated greater body weight adjusted loading rates in the dominant and non-dominant leg, respectively. Finally, no significant between-group differences were observed for body weight adjusted FM values.

Given that no significant between-group differences were detected in running speed and kinetic data were adjusted for body weight, validity of our data is given.

### Effects of genu varus on ground reaction forces

Our results are partly in accordance with the relevant literature with regards to the observed GRF values on the dominant and non-dominant limb during running at preferred speed [5,6]. In the present study, individuals with knee genu varus produced significantly higher body weight adjusted GRF values in the lateral direction (Fx) of the dominant limb and significantly higher body weight adjusted GRF values in the lateral and medial directions (Fx) of the non-dominant limb. Furthermore, there is a considerable variation both within and between groups in the pattern of medio-lateral GRF during the stance phase of running (see Fig 2).

Of note, a number of studies reported abnormal control of mediolateral motion in children and/or adolescents with cerebral palsy [31], hearing loss [32], down syndrome [33], and scoliosis [34]. It has been demonstrated that during walking at different velocities (i.e., 0.54; 0.75; 1.15; 1.56 m/s) the abductor, vasti, and plantarflexor muscles) make significantly larger contributions to mediolateral GRF compared to the contributions of passive dynamics (i.e., gravity and velocity-related forces) [35]. On average, the above reported muscles contributed 92% of the total mediolateral GRF over all examined walking speeds [35]. In this context, Liu et al. postulated that the abductors primarily support body weight by contributing a large medial GRF at all walking speeds, the vasti, gastrocnemius, and soleus influence both, forward progression and body weight support [36]. Also, it has been demonstrated that electromyographical activity of the biceps femoris muscle was significantly higher in boys with genu varus compared to healthy controls during the loading response phase of walking [14]. In the present study, we observed higher mediolateral GRF (Fx) in genu varus patients which is primarily caused by varus malalignment and to a lesser extent by muscular disorders [14]. In young individuals with varus malalignment, abnormally high knee internal rotation and hip external rotation moments were detected during walking which may increase the risk of sustaining knee injuries [6]. Apart from that, higher lateral (Fx) GRF were found in the present study in children with genu varus. It has previously been postulated for runners that high lateral GRF result in overpronation during running [37,38] which may again cause overuse syndromes of the leg and the knee joint [39]. If signs of abnormally high lateral GRF are observed in children, it is recommended to conduct preventive training programs (e.g., balance and/or strength training) that focuses on lower limb alignment and control mediolateral motion from an early age on [40]. Therefore, practitioners and therapists are advised to conduct balance and strength training to improve lower limb alignment and mediolateral control during dynamic movements in children with genu varus.

### Effects of genu varus on vertical loading rates

Our results are partly in accordance with the relevant literature with regards to the observed vertical loading rate values on the dominant and non-dominant limb [41,42]. Findings from the present study demonstrated greater body weight adjusted vertical loading rates in both, the dominant and non-dominant lower limbs in children with genu varus compared to healthy controls. Higher vertical loading rates in children with genu varus are most likely due to a shorter time to reach peak GRF (Table 3 and Fig 2).

It has been shown that repetitive impact loading results in subchondral bone microdamage associated with cartilage thinning [43]. Also, the first peak of vertical GRF curve has high frequency domains that are propagated through the lower limb and modulated by the active and passive structure of the lower extremities, such as the muscles, tendons, ligaments, bone, and cartilage. Impairments to the active (muscles) and passive structures (such as ligaments and etc) of the body may be associated with dysfunction in impact attenuation process. This dysfunction results in forces across the articular surfaces of the lower limb joints (i.e., knee) to be increased beyond the tissues' load-bearing capacity [44], especially the cartilage regions that are unaccustomed to such forces [45,46]. It has been suggested that the vertical loading rate is a useful variable for estimating the overload applied on the lower limb musculoskeletal tissues [47,48]. An average vertical loading rate greater than 70, 72, and 100 N/kg/s during running in runners has been associated with a risk of sustaining stress fractures [49,50], patellofemoral pain [41,51], and plantar fasciitis [42]. In the present study, genu varus children demonstrated vertical loading rates that were greater than 80 N/kg/s. Therefore, it can be postulated that children with genu varus are at risk of sustaining stress fractures [49,50] and patellofemoral pain syndrome [41,51].

### Effects of genu varus on free moment amplitudes

Our results are not in accordance with the literature in terms of the observed FM values on the dominant and non-dominant limbs [5,28,52]. Our study revealed no statistically significant between-group differences in body weight adjusted FM values during the stance phase of a running cycle. It has previously been demonstrated that knee varus malalignment results in larger endorotation of the foot and greater internal tibia rotation during the stance phase of walking [5]. Further, it has been demonstrated that runners with an injury history (tibial stress fracture and over pronation) showed higher free moment amplitudes compared to healthy (uninjured) runners [28,29,53]. This highlights the importance of assessing FM and thus biomechanical loading of the lower limbs in other populations. Nevertheless, our results did not demonstrate any significant differences in running FM peaks between genu varus group and healthy controls. However, as was illustrated in Fig 5, there was a considerable variation both within and between groups in the pattern of free moment during the stance phase of running. Moreover, as is typical in ensemble curves, the peaks are attenuated relative to the individual curves owing to between-group differences in TTP GRF values. The group average curves (Fig 5) illustrate the overall pattern of FM during stance phase of running, but as can be seen from the large spread demonstrated by the standard deviation in Table 4, there was considerable between subject data variability. The present study provides fundamental information regarding the characteristics of FM in healthy boys as well as boys with genu varus during the stance phase of running. As mentioned in a previous study [52], it seems that FM could be used to classify genu varus children into functional groups since it possesses a considerable inter-subject variability, a relationship to the mechanical demands put on the lower limb joints and potentially to injury risk. This is likely due to the longer support time during walking versus running.

A few limitations of this study warrant discussion. First, we assessed kinetic data only which is why it is not possible to deduce the underlying neuromuscular mechanisms responsible for the observed findings. Thus, further research is needed in this area. Second, we examined boys with genu varus versus age-matched healthy controls. Therefore, our findings should be carefully interpreted in girls and adolescents. More research is needed to confirm our findings for different population groups.

## Conclusions

A mechanical consequence of knee varus malalignment is a greater lateral (Fx) GRF amplitude, lower TTP three dimensional GRF components, greater vertical loading rates, and greater mediolateral impulses in both limbs during the stance phase of running. Of note, the genu varus compared to the control group did not show any differences in peak negative and positive FM. Due to the higher GRFs, higher loading rates, and impulses put upon the lower limbs of genu varus children, various invasive or non-invasive treatment strategies such as surgery, orthoses, or balance and strength training protocols should be considered to prevent joint degeneration in these young individuals.

## Supporting information

S1 File Minimal data set for Fig 2. (S1 Fig 2).
(XLSX)

S2 File Minimal data set for Fig 3 and Fig 4. (S2 Fig 4).
(XLSX)

S3 File Minimal data set for Fig 5. (S3 Fig 5).
(XLSX)
